# 1-(2,4-Dinitro­phen­yl)-3-phenyl-4-phenyl­sulfanyl-1*H*-pyrazole

**DOI:** 10.1107/S1600536812036914

**Published:** 2012-09-05

**Authors:** V. Susindran, S. Athimoolam, S. Asath Bahadur, R. Manikannan, S. Muthusubramanian

**Affiliations:** a Department of Physics, Kalasalingam University, Anand Nagar, Krishnan Koil 626 190, India; b Department of Physics, University College of Engineering Nagercoil, Anna University Chennai, Nagercoil 629 004, India; c Department of Organic Chemistry, Madurai Kamaraj University, Madurai 625 021, India

## Abstract

In the title mol­ecule, C_21_H_14_N_4_O_4_S, the pyrazole ring forms dihedral angles of 45.6 (1), 87.7 (1) and 27.4 (1)° with the phenyl, sulfur-substituted benzene and nitro-substituted benzene rings, respectively. In the crystal, mol­ecules are connected by weak C—H⋯O and C—H⋯N hydrogen bonds into layers parallel to (010).

## Related literature

For the pharmacological and medicinal properties of pyrazole compounds, see: Baraldi *et al.* (1998 ▶); Bruno *et al.* (1990 ▶); Chen & Li (1998 ▶); Cottineau *et al.* (2002 ▶); Londershausen (1996 ▶); Mishra *et al.* (1998 ▶); Smith *et al.* (2001 ▶).
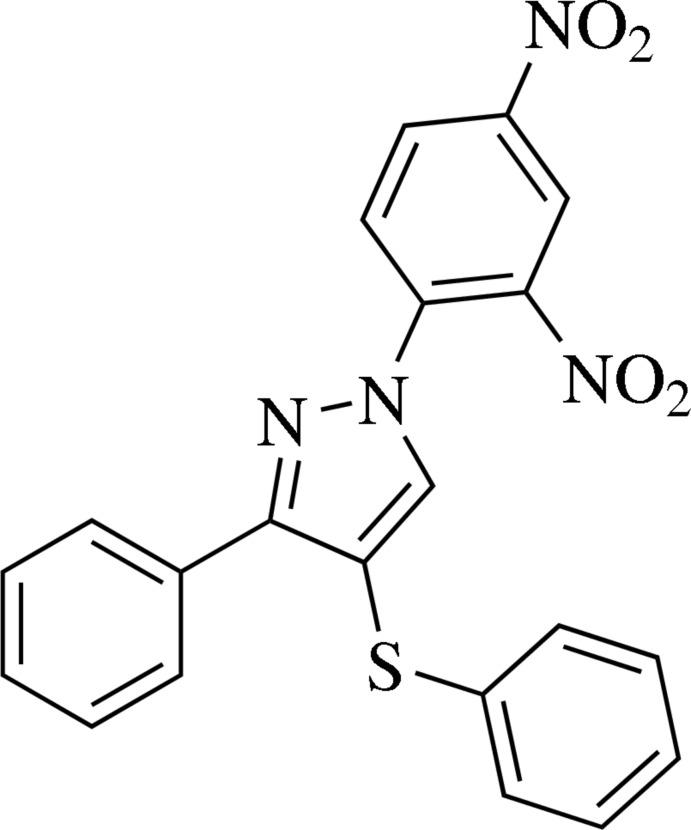


## Experimental

### 

#### Crystal data

C_21_H_14_N_4_O_4_S
*M* *_r_* = 418.42
Monoclinic, 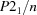

*a* = 7.3062 (4) Å
*b* = 26.5212 (13) Å
*c* = 10.3361 (5) Å
β = 104.012 (1)°
*V* = 1943.22 (17) Å^3^
*Z* = 4
Mo *K*α radiation
μ = 0.20 mm^−1^
*T* = 293 K
0.24 × 0.21 × 0.18 mm

#### Data collection

Bruker SMART APEX CCD diffractometer
18559 measured reflections
3426 independent reflections
3128 reflections with *I* > 2σ(*I*)
*R*_int_ = 0.021

#### Refinement

*R*[*F* ^2^ > 2σ(*F* ^2^)] = 0.035
*wR*(*F* ^2^) = 0.091
*S* = 1.04
3426 reflections
271 parameters
H-atom parameters constrained
Δρ_max_ = 0.27 e Å^−3^
Δρ_min_ = −0.14 e Å^−3^

### 

Data collection: *SMART* (Bruker, 2001 ▶); cell refinement: *SAINT* (Bruker, 2001 ▶); data reduction: *SAINT*; program(s) used to solve structure: *SHELXTL* (Sheldrick, 2008 ▶); program(s) used to refine structure: *SHELXTL*; molecular graphics: *PLATON* (Spek, 2009 ▶); software used to prepare material for publication: *SHELXTL*.

## Supplementary Material

Crystal structure: contains datablock(s) global, I. DOI: 10.1107/S1600536812036914/lh5484sup1.cif

Structure factors: contains datablock(s) I. DOI: 10.1107/S1600536812036914/lh5484Isup2.hkl

Supplementary material file. DOI: 10.1107/S1600536812036914/lh5484Isup3.cml

Additional supplementary materials:  crystallographic information; 3D view; checkCIF report

## Figures and Tables

**Table 1:** Hydrogen-bond geometry (Å, °)

*D*—H⋯*A*	*D*—H	H⋯*A*	*D*⋯*A*	*D*—H⋯*A*
C35—H35⋯O42^i^	0.93	2.58	3.452 (2)	157
C5—H5⋯N2^ii^	0.93	2.52	3.411 (2)	161

Symmetry codes: (i) 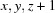; (ii) 
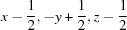
.

**Fig. 1.**
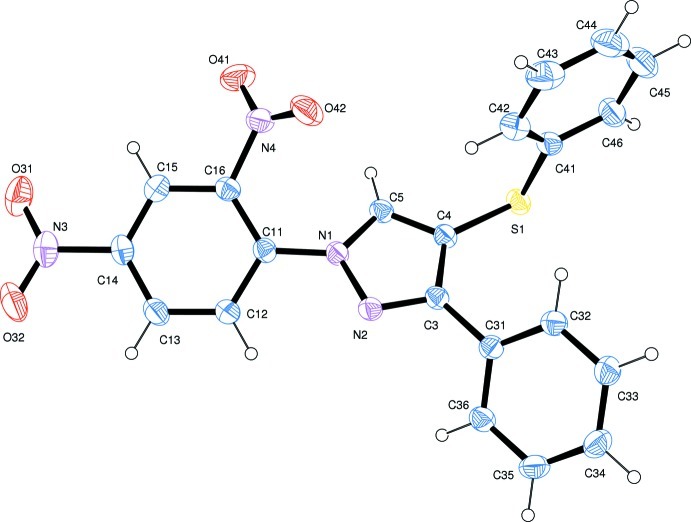
The molecular structure of the title compound with 50% probability displacement ellipsoids.

**Fig. 2.**
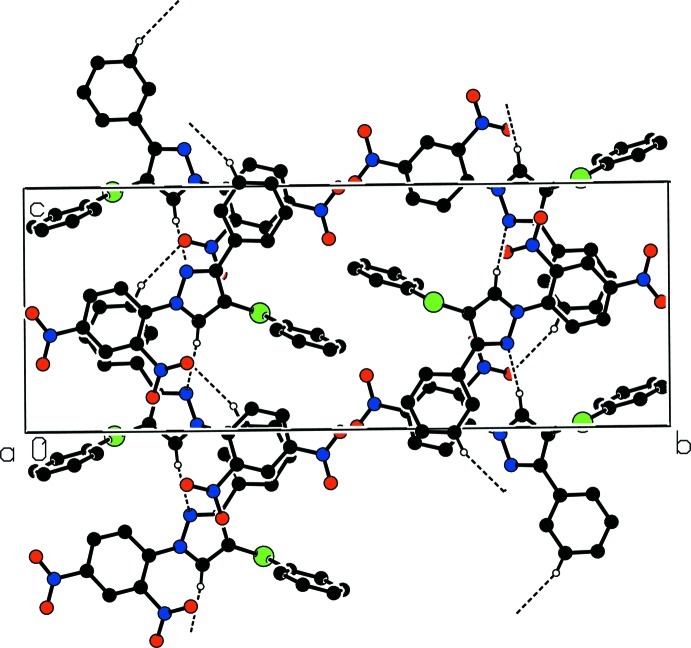
Part of the crystal structure with hydrogen bonds shown as dashed lines.

## supplementary crystallographic information

### Comment

Pyrazoles are a class of aromatic ring compounds and of the heterocyclic series characterized by a 5-membered ring structure composed of three carbon atoms and two nitrogen atoms in adjacent positions and to the unsubstituted parent compound. They can have pharmacological effects on humans and are classified as alkaloids although they are rare in nature. Pyrazole and its derivatives have successfully tested for antifungal (Chen & Li, 1998), antihistaminic (Mishra *et al.*,1998), anti-inflammatory (Smith *et al.*, 2001), antiarrhythmic and sedative (Bruno *et al.*, 1990), hypoglycemic (Cottineau *et al.*, 2002), antiviral (Baraldi *et al.*, 1998) and pesticidal (Londershausen, 1996) activities.

The molecular structure of the title compound is shown in Fig. 1. The pyrazole ring forms dihedral angles of 27.4 (1)° (with the C11-C16 ring), 45.6 (1)° (with the C31-C36 ring) and 87.7 (1)° (with the C41-C46 ring). In the nitrophenyl group, the nitro substituents are twisted from the benzen ring by 13.9 (2)° and 43.2 (1)°. In the crystal, molecules are connected by weak C—H···O and C—H···N hydrogen bonds into layers parallel to (010) (see Fig. 2).

### Experimental

A mixture of 1-phenyl-2-(phenylsulfanyl)-1-ethanone 1-(2,4-dinitrophenyl)hydrazone (0.001 mole) dissolved in dimethylforamide (5 ml) in a 30 ml conical flask was allowed to cool in ice with stirring. To this stirred solution, phosphorus oxychloride (0.008 mole) was added dropwise and the mixture was subjected to microwave irradiation for 30 sec. The reaction was monitored by TLC and after completion of the reaction, the reaction mixture was poured onto crushed ice. The solid was suction filtered and washed with plenty of water. The final product 1-(2,4-dinitrophenyl)-3-phenyl-4-(phenylsulfanyl)-1*H*-pyrazole was purified by column chromatography using petroleum ether-ethyl acetate as eluent. Colourless needles were grown over a period of a week from a solution of the title compound in dichlromethane.

### Refinement

All H atoms were positioned geometrically and refined using a riding model, with C—H = 0.93 Å and *U*_iso_(H) = 1.2 *U*_eq_(C).

### Crystal data

**Table:**

C_21_H_14_N_4_O_4_S	*F*(000) = 864
*M**_r_* = 418.42	*D*_x_ = 1.430 Mg m^−^^3^
Monoclinic, *P*2_1_/*n*	Mo *K*α radiation, λ = 0.71073 Å
Hall symbol: -P 2yn	Cell parameters from 4527 reflections
*a* = 7.3062 (4) Å	θ = 2.1–24.4°
*b* = 26.5212 (13) Å	µ = 0.20 mm^−^^1^
*c* = 10.3361 (5) Å	*T* = 293 K
β = 104.012 (1)°	Block, colourless
*V* = 1943.22 (17) Å^3^	0.24 × 0.21 × 0.18 mm
*Z* = 4	

### Data collection

**Table:**

Bruker SMART APEX CCD diffractometer	3128 reflections with *I* > 2σ(*I*)
Radiation source: fine-focus sealed tube	*R*_int_ = 0.021
Graphite monochromator	θ_max_ = 25.0°, θ_min_ = 1.5°
ω scans	*h* = −8→8
18559 measured reflections	*k* = −31→31
3426 independent reflections	*l* = −12→12

### Refinement

**Table:**

Refinement on *F*^2^	Primary atom site location: structure-invariant direct methods
Least-squares matrix: full	Secondary atom site location: difference Fourier map
*R*[*F*^2^ > 2σ(*F*^2^)] = 0.035	Hydrogen site location: inferred from neighbouring sites
*wR*(*F*^2^) = 0.091	H-atom parameters constrained
*S* = 1.04	*w* = 1/[σ^2^(*F*_o_^2^) + (0.0451*P*)^2^ + 0.5673*P*] where *P* = (*F*_o_^2^ + 2*F*_c_^2^)/3
3426 reflections	(Δ/σ)_max_ < 0.001
271 parameters	Δρ_max_ = 0.27 e Å^−^^3^
0 restraints	Δρ_min_ = −0.14 e Å^−^^3^

### Special details

**Table:**

Geometry. All e.s.d.'s (except the e.s.d. in the dihedral angle between two l.s. planes) are estimated using the full covariance matrix. The cell e.s.d.'s are taken into account individually in the estimation of e.s.d.'s in distances, angles and torsion angles; correlations between e.s.d.'s in cell parameters are only used when they are defined by crystal symmetry. An approximate (isotropic) treatment of cell e.s.d.'s is used for estimating e.s.d.'s involving l.s. planes.
Refinement. Refinement of *F*^2^ against ALL reflections. The weighted *R*-factor *wR* and goodness of fit *S* are based on *F*^2^, conventional *R*-factors *R* are based on *F*, with *F* set to zero for negative *F*^2^. The threshold expression of *F*^2^ > σ(*F*^2^) is used only for calculating *R*-factors(gt) *etc*. and is not relevant to the choice of reflections for refinement. *R*-factors based on *F*^2^ are statistically about twice as large as those based on *F*, and *R*- factors based on ALL data will be even larger.

### Fractional atomic coordinates and isotropic or equivalent isotropic displacement parameters (Å^2^)

**Table:**

	*x*	*y*	*z*	*U*_iso_*/*U*_eq_	
N1	0.41540 (17)	0.26466 (4)	0.02430 (12)	0.0380 (3)	
C11	0.4691 (2)	0.31285 (5)	−0.01058 (15)	0.0379 (3)	
C12	0.5120 (2)	0.34984 (6)	0.08788 (16)	0.0466 (4)	
H12	0.5059	0.3421	0.1745	0.056*	
C13	0.5635 (3)	0.39766 (6)	0.05891 (17)	0.0510 (4)	
H13	0.5969	0.4217	0.1259	0.061*	
C14	0.5647 (2)	0.40930 (6)	−0.07041 (17)	0.0458 (4)	
C15	0.5206 (2)	0.37446 (6)	−0.17104 (16)	0.0453 (4)	
H15	0.5197	0.3832	−0.2583	0.054*	
C16	0.4775 (2)	0.32614 (6)	−0.13962 (15)	0.0407 (3)	
N3	0.6219 (2)	0.46010 (6)	−0.10176 (19)	0.0597 (4)	
O31	0.5928 (2)	0.47181 (5)	−0.21916 (17)	0.0820 (5)	
O32	0.6962 (2)	0.48746 (5)	−0.00956 (17)	0.0849 (5)	
N4	0.4608 (2)	0.28911 (6)	−0.24824 (15)	0.0546 (4)	
O41	0.3765 (2)	0.30239 (6)	−0.35976 (13)	0.0816 (5)	
O42	0.5384 (2)	0.24840 (5)	−0.22055 (15)	0.0727 (4)	
N2	0.48161 (17)	0.24827 (4)	0.15338 (12)	0.0385 (3)	
C3	0.4084 (2)	0.20278 (5)	0.15590 (14)	0.0363 (3)	
C31	0.4517 (2)	0.17255 (5)	0.27946 (14)	0.0379 (3)	
C32	0.4992 (2)	0.12194 (6)	0.27580 (16)	0.0447 (4)	
H32	0.5016	0.1070	0.1948	0.054*	
C33	0.5428 (3)	0.09357 (6)	0.39095 (17)	0.0518 (4)	
H33	0.5753	0.0598	0.3875	0.062*	
C34	0.5382 (3)	0.11532 (7)	0.51093 (17)	0.0565 (5)	
H34	0.5673	0.0962	0.5886	0.068*	
C35	0.4907 (2)	0.16534 (7)	0.51629 (16)	0.0536 (4)	
H35	0.4879	0.1799	0.5977	0.064*	
C36	0.4472 (2)	0.19411 (6)	0.40134 (15)	0.0439 (4)	
H36	0.4149	0.2279	0.4055	0.053*	
C4	0.2922 (2)	0.18993 (5)	0.02874 (14)	0.0380 (3)	
S1	0.15552 (6)	0.135789 (14)	−0.01962 (4)	0.04436 (13)	
C41	0.3132 (2)	0.09507 (6)	−0.07662 (15)	0.0454 (4)	
C42	0.4896 (3)	0.10892 (7)	−0.08882 (18)	0.0576 (4)	
H42	0.5346	0.1413	−0.0656	0.069*	
C43	0.5999 (3)	0.07424 (9)	−0.1360 (2)	0.0716 (6)	
H43	0.7196	0.0834	−0.1437	0.086*	
C44	0.5341 (4)	0.02652 (9)	−0.1715 (2)	0.0768 (6)	
H44	0.6087	0.0035	−0.2034	0.092*	
C45	0.3590 (4)	0.01295 (8)	−0.1596 (2)	0.0745 (6)	
H45	0.3141	−0.0193	−0.1841	0.089*	
C46	0.2477 (3)	0.04661 (6)	−0.11152 (18)	0.0588 (5)	
H46	0.1291	0.0369	−0.1025	0.071*	
C5	0.2996 (2)	0.23037 (5)	−0.05062 (15)	0.0387 (3)	
H5	0.2367	0.2338	−0.1399	0.046*	

### Atomic displacement parameters (Å^2^)

**Table:**

	*U*^11^	*U*^22^	*U*^33^	*U*^12^	*U*^13^	*U*^23^
N1	0.0427 (7)	0.0330 (6)	0.0352 (6)	−0.0002 (5)	0.0035 (5)	−0.0003 (5)
C11	0.0379 (8)	0.0341 (7)	0.0398 (8)	0.0013 (6)	0.0060 (6)	−0.0006 (6)
C12	0.0599 (10)	0.0391 (8)	0.0402 (9)	−0.0022 (7)	0.0109 (7)	−0.0027 (7)
C13	0.0608 (10)	0.0377 (8)	0.0527 (10)	−0.0039 (7)	0.0099 (8)	−0.0083 (7)
C14	0.0438 (9)	0.0337 (8)	0.0589 (10)	−0.0004 (6)	0.0107 (7)	0.0027 (7)
C15	0.0428 (8)	0.0487 (9)	0.0446 (9)	−0.0008 (7)	0.0108 (7)	0.0065 (7)
C16	0.0396 (8)	0.0420 (8)	0.0397 (8)	−0.0025 (6)	0.0082 (6)	−0.0050 (6)
N3	0.0548 (9)	0.0419 (8)	0.0835 (12)	0.0016 (7)	0.0189 (8)	0.0118 (8)
O31	0.0934 (11)	0.0606 (9)	0.0907 (11)	−0.0049 (8)	0.0199 (9)	0.0289 (8)
O32	0.1012 (12)	0.0454 (8)	0.1062 (12)	−0.0240 (8)	0.0213 (9)	−0.0121 (8)
N4	0.0597 (9)	0.0609 (10)	0.0481 (9)	−0.0199 (7)	0.0225 (7)	−0.0143 (7)
O41	0.1011 (11)	0.1023 (12)	0.0392 (7)	−0.0322 (9)	0.0129 (7)	−0.0093 (7)
O42	0.0884 (10)	0.0552 (8)	0.0825 (10)	−0.0049 (7)	0.0366 (8)	−0.0259 (7)
N2	0.0425 (7)	0.0372 (7)	0.0334 (6)	−0.0002 (5)	0.0043 (5)	−0.0010 (5)
C3	0.0366 (7)	0.0344 (7)	0.0371 (8)	0.0020 (6)	0.0074 (6)	−0.0021 (6)
C31	0.0361 (7)	0.0401 (8)	0.0359 (8)	−0.0031 (6)	0.0056 (6)	−0.0009 (6)
C32	0.0518 (9)	0.0427 (8)	0.0379 (8)	0.0027 (7)	0.0076 (7)	−0.0020 (6)
C33	0.0608 (10)	0.0427 (9)	0.0481 (10)	0.0021 (8)	0.0057 (8)	0.0061 (7)
C34	0.0648 (11)	0.0596 (11)	0.0404 (9)	−0.0086 (9)	0.0032 (8)	0.0107 (8)
C35	0.0606 (10)	0.0646 (11)	0.0345 (8)	−0.0125 (9)	0.0097 (7)	−0.0057 (7)
C36	0.0448 (9)	0.0433 (8)	0.0422 (8)	−0.0065 (7)	0.0078 (7)	−0.0064 (7)
C4	0.0390 (8)	0.0343 (7)	0.0384 (8)	−0.0003 (6)	0.0052 (6)	−0.0029 (6)
S1	0.0456 (2)	0.0389 (2)	0.0466 (2)	−0.00736 (16)	0.00724 (17)	−0.00609 (16)
C41	0.0594 (10)	0.0408 (8)	0.0329 (8)	0.0021 (7)	0.0050 (7)	−0.0023 (6)
C42	0.0671 (12)	0.0552 (10)	0.0534 (10)	−0.0013 (9)	0.0199 (9)	−0.0060 (8)
C43	0.0754 (13)	0.0867 (15)	0.0574 (12)	0.0130 (11)	0.0250 (10)	−0.0043 (11)
C44	0.1021 (18)	0.0722 (14)	0.0556 (12)	0.0298 (13)	0.0180 (11)	−0.0104 (10)
C45	0.1061 (18)	0.0479 (11)	0.0631 (12)	0.0123 (11)	0.0081 (12)	−0.0130 (9)
C46	0.0742 (12)	0.0433 (9)	0.0542 (10)	−0.0012 (8)	0.0061 (9)	−0.0061 (8)
C5	0.0406 (8)	0.0367 (7)	0.0353 (8)	0.0014 (6)	0.0025 (6)	−0.0025 (6)

### Geometric parameters (Å, º)

**Table:**

N1—C5	1.3508 (18)	C32—H32	0.9300
N1—N2	1.3749 (17)	C33—C34	1.375 (3)
N1—C11	1.4093 (18)	C33—H33	0.9300
C11—C12	1.393 (2)	C34—C35	1.376 (3)
C11—C16	1.395 (2)	C34—H34	0.9300
C12—C13	1.376 (2)	C35—C36	1.383 (2)
C12—H12	0.9300	C35—H35	0.9300
C13—C14	1.374 (2)	C36—H36	0.9300
C13—H13	0.9300	C4—C5	1.359 (2)
C14—C15	1.370 (2)	C4—S1	1.7515 (14)
C14—N3	1.470 (2)	S1—C41	1.7805 (17)
C15—C16	1.377 (2)	C41—C42	1.375 (2)
C15—H15	0.9300	C41—C46	1.388 (2)
C16—N4	1.474 (2)	C42—C43	1.387 (3)
N3—O32	1.216 (2)	C42—H42	0.9300
N3—O31	1.220 (2)	C43—C44	1.372 (3)
N4—O41	1.220 (2)	C43—H43	0.9300
N4—O42	1.221 (2)	C44—C45	1.363 (3)
N2—C3	1.3226 (18)	C44—H44	0.9300
C3—C4	1.423 (2)	C45—C46	1.378 (3)
C3—C31	1.476 (2)	C45—H45	0.9300
C31—C32	1.389 (2)	C46—H46	0.9300
C31—C36	1.391 (2)	C5—H5	0.9300
C32—C33	1.379 (2)		
			
C5—N1—N2	111.59 (11)	C34—C33—C32	119.89 (16)
C5—N1—C11	129.66 (12)	C34—C33—H33	120.1
N2—N1—C11	118.72 (11)	C32—C33—H33	120.1
C12—C11—C16	117.79 (14)	C33—C34—C35	120.16 (16)
C12—C11—N1	118.62 (13)	C33—C34—H34	119.9
C16—C11—N1	123.57 (13)	C35—C34—H34	119.9
C13—C12—C11	120.99 (15)	C34—C35—C36	120.34 (15)
C13—C12—H12	119.5	C34—C35—H35	119.8
C11—C12—H12	119.5	C36—C35—H35	119.8
C14—C13—C12	119.03 (15)	C35—C36—C31	120.03 (15)
C14—C13—H13	120.5	C35—C36—H36	120.0
C12—C13—H13	120.5	C31—C36—H36	120.0
C15—C14—C13	122.07 (15)	C5—C4—C3	105.46 (12)
C15—C14—N3	118.56 (15)	C5—C4—S1	125.31 (11)
C13—C14—N3	119.32 (15)	C3—C4—S1	129.16 (11)
C14—C15—C16	118.29 (15)	C4—S1—C41	102.81 (7)
C14—C15—H15	120.9	C42—C41—C46	119.70 (17)
C16—C15—H15	120.9	C42—C41—S1	124.36 (13)
C15—C16—C11	121.73 (14)	C46—C41—S1	115.94 (14)
C15—C16—N4	114.95 (14)	C41—C42—C43	119.49 (18)
C11—C16—N4	123.04 (14)	C41—C42—H42	120.3
O32—N3—O31	124.40 (16)	C43—C42—H42	120.3
O32—N3—C14	118.10 (16)	C44—C43—C42	120.7 (2)
O31—N3—C14	117.51 (16)	C44—C43—H43	119.7
O41—N4—O42	125.20 (16)	C42—C43—H43	119.7
O41—N4—C16	117.19 (16)	C45—C44—C43	119.7 (2)
O42—N4—C16	117.54 (15)	C45—C44—H44	120.2
C3—N2—N1	104.98 (11)	C43—C44—H44	120.2
N2—C3—C4	110.73 (12)	C44—C45—C46	120.7 (2)
N2—C3—C31	120.64 (12)	C44—C45—H45	119.7
C4—C3—C31	128.63 (13)	C46—C45—H45	119.7
C32—C31—C36	118.85 (14)	C45—C46—C41	119.8 (2)
C32—C31—C3	120.26 (13)	C45—C46—H46	120.1
C36—C31—C3	120.89 (13)	C41—C46—H46	120.1
C33—C32—C31	120.73 (15)	N1—C5—C4	107.22 (13)
C33—C32—H32	119.6	N1—C5—H5	126.4
C31—C32—H32	119.6	C4—C5—H5	126.4
			
C5—N1—C11—C12	150.11 (15)	N2—C3—C31—C36	45.2 (2)
N2—N1—C11—C12	−27.9 (2)	C4—C3—C31—C36	−135.11 (16)
C5—N1—C11—C16	−28.3 (2)	C36—C31—C32—C33	−0.6 (2)
N2—N1—C11—C16	153.72 (14)	C3—C31—C32—C33	178.96 (15)
C16—C11—C12—C13	−0.9 (2)	C31—C32—C33—C34	0.4 (3)
N1—C11—C12—C13	−179.36 (15)	C32—C33—C34—C35	−0.2 (3)
C11—C12—C13—C14	2.6 (3)	C33—C34—C35—C36	0.0 (3)
C12—C13—C14—C15	−1.5 (3)	C34—C35—C36—C31	−0.2 (2)
C12—C13—C14—N3	−179.20 (15)	C32—C31—C36—C35	0.4 (2)
C13—C14—C15—C16	−1.3 (2)	C3—C31—C36—C35	−179.10 (14)
N3—C14—C15—C16	176.38 (14)	N2—C3—C4—C5	−0.01 (17)
C14—C15—C16—C11	3.1 (2)	C31—C3—C4—C5	−179.74 (14)
C14—C15—C16—N4	−170.88 (14)	N2—C3—C4—S1	−176.96 (11)
C12—C11—C16—C15	−2.1 (2)	C31—C3—C4—S1	3.3 (2)
N1—C11—C16—C15	176.35 (14)	C5—C4—S1—C41	91.96 (14)
C12—C11—C16—N4	171.45 (14)	C3—C4—S1—C41	−91.65 (15)
N1—C11—C16—N4	−10.1 (2)	C4—S1—C41—C42	−6.10 (16)
C15—C14—N3—O32	−165.63 (16)	C4—S1—C41—C46	174.66 (13)
C13—C14—N3—O32	12.1 (2)	C46—C41—C42—C43	0.0 (3)
C15—C14—N3—O31	13.8 (2)	S1—C41—C42—C43	−179.18 (14)
C13—C14—N3—O31	−168.44 (17)	C41—C42—C43—C44	0.5 (3)
C15—C16—N4—O41	−42.7 (2)	C42—C43—C44—C45	−0.3 (3)
C11—C16—N4—O41	143.32 (16)	C43—C44—C45—C46	−0.4 (3)
C15—C16—N4—O42	134.48 (16)	C44—C45—C46—C41	0.9 (3)
C11—C16—N4—O42	−39.4 (2)	C42—C41—C46—C45	−0.7 (3)
C5—N1—N2—C3	1.50 (16)	S1—C41—C46—C45	178.53 (15)
C11—N1—N2—C3	179.83 (12)	N2—N1—C5—C4	−1.54 (17)
N1—N2—C3—C4	−0.88 (16)	C11—N1—C5—C4	−179.63 (14)
N1—N2—C3—C31	178.87 (12)	C3—C4—C5—N1	0.92 (16)
N2—C3—C31—C32	−134.32 (15)	S1—C4—C5—N1	178.02 (11)
C4—C3—C31—C32	45.4 (2)		

### Hydrogen-bond geometry (Å, º)

**Table:**

*D*—H···*A*	*D*—H	H···*A*	*D*···*A*	*D*—H···*A*
C35—H35···O42^i^	0.93	2.58	3.452 (2)	157
C5—H5···N2^ii^	0.93	2.52	3.411 (2)	161

Symmetry codes: (i) *x*, *y*, *z*+1; (ii) *x*−1/2, −*y*+1/2, *z*−1/2.
